# Price of Nitazenes for Sale on Cryptomarkets and Surface Web Shops

**DOI:** 10.1111/dar.70202

**Published:** 2026-07-08

**Authors:** Nicola Man, Monica J. Barratt, Raimondo Bruno, Connor Graham, Vandit Sadaphale, Amy Peacock, Rachel Sutherland

**Affiliations:** ^1^ National Drug and Alcohol Research Centre, UNSW Sydney Sydney Australia; ^2^ National Drug Research Institute and enAble Institute, Curtin University Perth Australia; ^3^ Harm and Risk Reduction Program, Burnet Institute Melbourne Australia; ^4^ School of Psychological Sciences, University of Tasmania Hobart Australia

**Keywords:** cryptomarkets, drug markets, nitazenes, novel synthetic opioids

## Abstract

**Introduction:**

Nitazenes are potent synthetic opioids increasingly detected in illicit drug markets in Australia and globally. Little is known about the pricing of nitazenes, which can influence patterns of use and accessibility. This study examines the price of nitazenes listed for sale via cryptomarkets and surface web shops.

**Methods:**

We collected nitazene listings on English‐language cryptomarkets twice monthly from October 2021 to September 2024. Prices per gram (US dollars) were analysed using a multilevel generalised additive model, accounting for nitazene type, quantity, region of origin and delivery availability to Australia. Surface web shops were identified in March 2024 using the search query ‘buy [specific nitazene] Australia’, with data analysed descriptively.

**Results:**

A total of 10,749 pricing observations of nitazene powder were identified across 29 cryptomarkets; 10 surface web shops selling nitazenes were also found. Isotonitazene had the highest median price per gram ($80 USD) among the most frequently listed nitazenes. Cryptomarket prices were significantly higher for listings shipping to Australia and originating from regions outside Asia, although there was variation by nitazene type. Cryptomarket prices trended upwards from 2024 onwards. Substantial quantity discounts were observed across nitazene types. Surface web shop prices were consistently lower than cryptomarkets.

**Discussion and Conclusions:**

Nitazenes are available at relatively low prices across cryptomarkets and surface web shops. Given that price is a key determinant of drug‐related harm, these findings reinforce the need to monitor online markets for their role in nitazene supply and to expand harm reduction efforts addressing the risks from these substances.

## Key Point Summary

- Nitazenes are available at a relatively low price across cryptomarkets and surface web shops.
- Prices were higher for listings shipping to Australia and rose from 2024.
- Bulk discounts were common, lowering per‐gram costs.
- Surface web shops offered cheaper prices than cryptomarkets.
- Monitoring online marketplaces is critical to understanding shifts in drug supply.

## 1 Introduction

Nitazenes are a class of synthetic 2‐benzyl benzimidazole opioids, originally developed as analgesics but never approved for clinical use due to their high‐risk profile [1]. While similar in effect to morphine, some nitazene compounds exhibit markedly greater potency [2, 3]. For example, metonitazene has demonstrated potency comparable to fentanyl and other nitazenes (e.g., protonitazene, isotonitazene and etonitazene) have shown even greater potency. However, data on relative potency in humans remain limited [2, 4]. First detected in 1999 in forensic laboratories in the United States [5], nitazenes made a resurgence in 2019 in illicit drug markets in Europe [2] and North America [6]. Nitazenes have since been identified in over 30 countries, with 34 distinct variants reported to the United Nations Office on Drugs and Crime Early Warning Advisory as of February 2026 [7]. While a number of nitazenes have been placed under international and national drug control [8], rising hospitalisations and deaths relating to these drugs have been reported in Europe [9], the United Kingdom [4, 10, 11], North America [12] and Australia [13, 14, 15]. Emerging evidence suggests these harms are driven, at least in part, by unintentional exposure through substitution or adulteration of other illicit drugs [13, 14, 15].

Despite their increasing availability, there is no published research that has examined the pricing of nitazenes in offline (street) or online markets. Illicit drug prices can vary substantially, even over short periods and are strongly linked with patterns of use and harm [16]. For example, a recent review of price elasticity in illicit heroin markets found that a 10% price increase typically resulted in a 9% reduction in use [17]. Pricing studies also offer insight into market dynamics, with prior research showing price differences are largely driven by non‐material costs (e.g., risk of criminal sanctions), as well as the impacts of supply‐reduction efforts (e.g., law enforcement).

The nature of nitazene involvement in illicit drug markets presents particular challenges for measuring street‐level prices. As aforementioned, nitazenes are often substituted for or used to adulterate, other illicit drugs [18, 19]. This also results in unwitting purchase and use [13], limiting the reliability of self‐reported prices by consumers. Alternative methods, such as undercover police reporting of purchase price, have been shown to be subject to significant bias for other illicit drugs [20].

In contrast, analysis of online illicit drug markets has yielded valuable supply‐side insights on pricing of illicit drugs [20, 21] and particularly new psychoactive substances [22, 23]. Since the emergence of Silk Road in 2011 [24], there has been rapid growth in cryptomarkets facilitating the trade of illicit goods and services, particularly illegal drugs [25, 26]. Illicit drugs are also available via surface web shops [27], which are accessible via standard internet browsers [28]. Previous studies have documented availability of products marketed explicitly as nitazenes for purchase on these platforms [29, 30, 31, 32]; understanding their pricing in these settings can offer insight into their economic accessibility and potential for wider uptake. Such a study could reveal differences in pricing over time, between countries, across nitazene types and at varying purchase quantities, identifying market conditions that may influence use.

In order to address this knowledge gap, this study used data collected from cryptomarkets and surface web shops to examine online pricing of nitazenes, with a specific focus on the four key nitazenes identified in Australian toxicology data and drug alerts at that time (i.e., isotonitazene, metonitazene, etonitazene and protonitazene) [15, 33]. Given availability of detailed time series data from cryptomarkets, pricing on this platform was also compared by time, delivery to Australia, geographical region of origin and quantity.

## 2 Methods

### 2.1 Design

We collected and analysed two forms of observational pricing data from online markets selling illicit drugs: (i) nitazene powder listings on cryptomarkets; and (ii) nitazene listings on surface web shops identified using a search engine. The study is reported according to STROBE guidelines (see Appendix I, supporting information). Ethical approval was provided by the UNSW Human Research Ethics Committee (HC220754).

### 2.2 Data Sources

#### 2.2.1 Cryptomarkets

Cryptomarkets were identified via the Dread forum on the darknet and other online sources (e.g., TorTaxi). Markets were eligible for inclusion if they had more than one vendor, vendors delivering from multiple countries, listings displayed in English and over 100 drug listings (*n* = 34 markets; see Appendix II for market names). Searches for new cryptomarkets occurred approximately quarterly or more often when major markets closed. Data on drug listings were collected twice monthly from October 2021 to September 2024 as part of the Drugs and New Technologies project [34]. Data were gathered from indexed pages listing all advertised drugs using custom Python web crawling programs developed for each market; individual listing or vendor pages were not scraped. Relevant text (e.g., drug listing title) was extracted using the HTML scraping tool Beautiful Soup or Scrapy.

Names of nitazenes were identified from published journal articles and other online data sources [1, 35] to compile a list of potential terms and substrings for identifying nitazenes in extracted data. Initial identification involved listing titles matches with the substrings (e.g., ‘azene’, ‘pyne’, ‘pipne’, ‘itaze’, ‘etazen’, ‘dazen’, ‘benzimidazole’). Vague substrings were included to capture possible misspellings. These matched terms were then reviewed to create a refined list of specific identifier terms including common misspellings. Listings were confirmed as potentially selling nitazenes only if they matched to this list of specific nitazene terms, ensuring exclusion of listings for other drugs containing similar substrings (see Appendix II for terms). Types of nitazenes were identified from the listing title, noting multiple nitazenes could be identified per listing (see Appendix II for nitazene types). Only listings advertising a single nitazene for sale were included in this study. While this could theoretically encompass listings for products containing both a nitazene and another substance (e.g., heroin adulterated with a nitazene), no such listings were identified.

The form of the nitazene was categorised into ‘powder’, ‘pill/tablet/press/capsule’, ‘liquid’, ‘blotter’ and ‘unknown’ through identification by terms in the listing title or in the quantity fields (see Appendix II for form classification). Where a particular form could not be identified and the quantity was identified as a weight quantity (e.g., ‘grams’, ‘mg’, ‘kg’) the listing was assumed to be for the powder form of the drug.

A separate observation was created for each quantity variant in each listing to create the dataset of price observations. Price in USD per gram was computed using the currency conversion rates listed on the cryptomarket website or as derived from the European Central Bank for the date of snapshot using the CurrencyConverter package in Python [36]. The price observations were deduplicated by market, vendor name, listing title, country of origin (or region if unspecified), availability for delivery to Australia, snapshot period, quantity and price per gram. Based on price data extracted from surface web listings, listings with prices ≤ 1 USD per gram were considered implausible outliers and excluded from analysis. Similarly, based on our review of prices from licit research chemical shops, listings with prices ≥ 500 USD per gram were also excluded.

Availability for delivery to Australia was identified where the listing specified delivery to Australia, Oceania or worldwide. Due to varying platform functionalities and requirements, some listings lacked geographical destination or origin data. To address this, missing delivery information was inferred from other listings by the same vendor when over 95% of their listings consistently indicated either availability or non‐availability for delivery to Australia. Missing data on region (and country) of origin was similarly populated.

#### 2.2.2 Surface Web Shops

The search phrase ‘buy [specific nitazene] Australia’ was entered into a common online search engine to find surface web shops advertising sale of isotonitazene, metonitazene, etonitazene and protonitazene, the four key nitazenes identified in Australian toxicology data and drug alerts at that time [15, 33], in any form. Searches were conducted in March 2024. The top 60 search hits for each of the four key nitazenes (240 in total) in Australia were collated. Online shops appearing in two or more hits were selected for data collection, as these were considered more visible and therefore more likely to be used. The minimum and maximum quantities available for purchase and their respective prices were extracted from all listings advertising sale of any of these four nitazenes.

### 2.3 Statistical Analysis

#### 2.3.1 Analysis of Price of Nitazenes on the Cryptomarket

The price per gram was analysed in a multi‐level generalised additive model using the log‐gamma distribution. Vendor and market were random effects and the following were fixed effects: availability for delivery to Australia, geographical region of origin, the quantity in grams offered for purchase, the type of nitazene being listed (nitazenes with < 100 rows of price data were aggregated into an ‘others’ category). The time of snapshot was fitted using the default thin‐plate regression spline. Given the non‐linear relationship of quantity with price, only the common quantity variants with ≥ 100 rows of price data (i.e., 1, 2, 5, 10, 25, 50, 100, 250, 500 and 1000 g) were included so that the quantity in grams could be analysed as a categorical variable. Quantities less than 1 g were also excluded because of ambiguity in the weight units (e.g., mg versus mcg) and these small quantities were relatively uncommon (see Section 3 below). The most frequent category was chosen as the reference category for the categorical variables, except for nitazene type. Isotonitazene was chosen as the reference category for nitazene type as it was the most frequently available nitazene in the final year of study, as well as the most consistently available nitazene over the 3‐year study period. The median and the 1st and 3rd quartiles of price per gram are also presented for the categorical variables fitted as fixed effects in the model.

The price per gram for the four key nitazenes identified in Australia was further analysed in another multi‐level generalised additive model (also using the log‐gamma distribution) by fitting an interaction effect for each of these nitazenes and all other nitazenes with their availability for delivery to Australia, geographical region of origin, quantity offered for purchase and the time of snapshot using the thin‐plate regression spline. Etonitazene was grouped with other nitazenes because it had too few pricing observations in the cryptomarket data. The results are presented with each of the key nitazenes as a stratum. The median price per gram and the 1st and 3rd quartiles of the key nitazenes (excluding etonitazene) are also presented by each of the quantities in grams being offered, as well as by availability for delivery to Australia, geographical region of origin and the year of the snapshot split by quantity offered for purchase (1–10 g versus > 10 g). Ten grams was chosen as the cut‐off as it reflected the upper limit of the minimum weight available for purchase on the surface web shops.

The R package *mgcv* (version 1.9) was used to perform the regression analysis and to check model assumptions (e.g., normality of residuals, homogeneity of variance, number of knots for the spline) [37]. Results from the model are presented as price ratios. A price ratio, *y*, of a category means that it was *y* times that of the reference category, that is, a price ratio *y* < 1 means that it was *y* times cheaper while *y* > 1 means it was *y* times more expensive, as compared with the reference category.

#### 2.3.2 Analysis of Price of the Four Key Nitazenes on Surface Web Shops

For the price per gram with their respective minimum and maximum quantity, the median and 1st and 3rd quartiles of price per gram in USD for each of the four key nitazenes and for each specific quantity (e.g., 5 g of any of the four nitazenes) were computed across all the shops. We computed the median and 1st and 3rd quartiles of price per gram for each specific quantity to capture how pricing varied by purchase size. As data were collected at a single time point and limited contextual data were presented (e.g., country of origin), descriptive data only are presented.

## 3 Results

### 3.1 Sample Characteristics

#### 3.1.1 Nitazenes on Cryptomarkets

Of the 34 cryptomarkets included in this study (October 2021 to September 2024), 29 of them had at least one nitazene listing. A total of 18,206 price observations from 13,701 listings selling nitazenes were identified in this period: 18,050 of these identified one specific nitazene (98.2%) and 16,669 of the single nitazene listings were listed in powder form (87.4%). A total of 12,100 observations remained after deduplication, of which 115 observations did not have valid data on quantity (e.g., missing or units of measurement could not be found), 13 observations did not have price for computing price per gram, 583 observations sold in < 1 g quantities (the most frequent quantity < 1 g was 0.5 g which had 191 observations) and 592 had fewer than 100 observations for a quantity. Of the 10,797 observations at the last stage of exclusion, 48 (0.4%) price outliers were excluded, of which six were extreme outliers ≥ 500 USD per gram, 19 were advertised at a total cost of 1.02 USD or less and the majority of the other 23 observations had ambiguous data on quantities for sale. There were 10,749 pricing observations in the final sample for the cryptomarket price analysis (see Figure B in Appendix S1 for flowchart of data exclusions). The most frequently available nitazenes in powder form were metonitazene (18.8%; *n* = 2023), isotonitazene (18.5%; *n* = 1990) and protonitazene (16.9%; *n* = 1815), followed by etodesnitazene (5.7%; *n* = 617).

#### 3.1.2 Nitazene Listings on Surface Web Shops

We identified 24 shops on the surface web selling nitazenes; 11 shops had a total of two or more hits in the searches conducted in the middle of March 2024. One shop could not be accessed when listings and delivery information were collected in early April 2024. Of the 10 shops studied for the present work, 7, 7, 5 and 6 shops sold isotonitazene, protonitazene, metonitazene and etonitazene, respectively.

### 3.2 Pricing on Cryptomarkets

*Nitazene type*. The median price of isotonitazene in the study period was 80.00 USD per gram (Table 1 and Table E in Appendix III). All other nitazenes were significantly cheaper compared to isotonitazene, ranging from a price ratio of 0.74 (95% confidence interval [CI] = 0.73, 0.76) for protonitazene to 0.50 (95% CI = 0.49, 0.52) for N‐desethyl etonitazene (Table 1). Note that the median prices may not align with the price ratios because the regression model adjusted the estimated price ratio for other explanatory variables in the model.

**TABLE 1:** Price of nitazene powders available for purchase on cryptomarkets.

	Freq.	Median USD per gram (Q1, Q3)^a^	Price ratio (95% CI)^b^
Nitazene name			
Metonitazene	2023	40.00 (18.85, 64.00)	0.56 (0.55, 0.57)***
Isotonitazene	1990	80.00 (52.00, 120.00)	Ref
Protonitazene	1815	38.00 (25.20, 70.00)	0.74 (0.73, 0.76)***
Etomethazene	798	64.41 (31.20, 97.52)	0.62 (0.61, 0.64)***
Butonitazene	744	30.00 (20.00, 55.00)	0.66 (0.64, 0.68)***
Protonitazepyne	663	34.80 (24.00, 56.00)	0.65 (0.63, 0.67)***
Etodesnitazene	617	88.81 (40.00, 150.00)	0.64 (0.62, 0.66)***
N‐desethyl isotonitazene	537	34.00 (21.00, 50.00)	0.53 (0.52, 0.54)***
Etonitazepyne	456	60.00 (29.00, 90.00)	0.81 (0.78, 0.84)***
Metonitazepyne	391	25.60 (20.00, 52.00)	0.60 (0.57, 0.62)***
N‐desethyl etonitazene	349	38.00 (22.00, 54.00)	0.50 (0.49, 0.52)***
Fluonitazene	233	34.00 (30.00, 58.00)	0.62 (0.59, 0.65)***
Others^c^	133	70.00 (60.00, 100.00)	0.71 (0.67, 0.76)***
Delivery to Australia			
Yes	9551	50.00 (26.00, 79.46)	Ref
No/unknown	1198	38.00 (20.00, 60.09)	0.87 (0.83, 0.91)***
Region of origin			
Asia	7315	38.00 (24.00, 68.00)	Ref
North America	1678	77.30 (46.00, 114.16)	1.04 (1.01, 1.07)**
Europe	605	87.50 (50.00, 124.42)	1.25 (1.20, 1.31)***
Oceania	405	64.00 (31.20, 216.98)	1.10 (1.06, 1.14)***
Unknown	746	46.00 (20.00, 70.00)	1.17 (1.12, 1.23)***
Quantity for sale (g)			
1	689	124.42 (90.00, 215.64)	1.34 (1.31, 1.38)***
2	282	95.00 (87.50, 225.00)	1.19 (1.14, 1.23)***
5	2043	74.00 (62.00, 90.00)	Ref
10	1814	60.00 (42.00, 75.00)	0.77 (0.75, 0.78)***
25	1331	36.00 (28.00, 56.00)	0.53 (0.52, 0.54)***
50	1128	32.00 (24.00, 52.00)	0.46 (0.45, 0.47)***
100	1154	29.00 (19.00, 48.50)	0.38 (0.37, 0.38)***
250	697	25.20 (21.60, 34.80)	0.30 (0.29, 0.30)***
500	790	21.00 (17.00, 29.00)	0.24 (0.24, 0.25)***
1000	821	16.50 (14.00, 21.00)	0.19 (0.18, 0.19)***

*Note:* Total number of listings included is *N* = 10,749. Listings without a single nitazene identified were excluded.

Abbreviation: CI, confidence interval.

^a^ Q1 = 1st quartile; Q3 = 3rd quartile.

^b^ ***indicates statistical significance at *p* < 0.001 and **indicates statistical significance at *p* < 0.01.

^c^ Nitazenes with < 100 rows of price data were aggregated into “Others”.

#### 3.2.1 Delivery to Australia

The price of nitazenes was cheaper for listings that did not offer delivery to Australia (median 50.00 USD per gram) as compared to those that did offer Australian delivery (median 38.00 USD per gram; price ratio = 0.87; 95% CI = 0.83, 0.91) (Table 1). This was similar for protonitazene listings (price ratio = 0.77; 95% CI = 0.71, 0.83) and ‘other nitazenes’ listings (price ratio = 0.88; 95% CI = 0.84, 0.92), while isotonitazene listings that were not available for delivery to Australia were more expensive (price ratio = 1.08; 95% CI = 1.00, 1.17; Table 2). There was no significant difference for metonitazene listings.

**TABLE 2:** Association of price of the key nitazenes identified in Australia on cryptomarkets with availability for delivery to Australia, region of origin and quantity for sale.

	Price ratio (95% CI)			
	Metonitazene	Isotonitazene	Protonitazene	Other nitazenes
Delivery to Australia				
Yes	ref	ref	ref	ref
No	1.06 (0.98, 1.15)	1.08 (1.00, 1.17)*	0.77 (0.71, 0.83)***	0.88 (0.84, 0.92)***
Region of origin				
Asia	ref	ref	ref	ref
North America	1.22 (1.15, 1.29)***	0.91 (0.88, 0.93)***	1.46 (1.37, 1.56)***	1.20 (1.14, 1.27)***
Europe	1.37 (1.27, 1.47)***	1.81 (1.60, 2.04)***	1.51 (1.33, 1.72)***	1.17 (1.10, 1.25)***
Oceania	1.05 (0.97, 1.14)	0.80 (0.70, 0.91)***	1.10 (0.95, 1.28)	1.18 (1.13, 1.22)***
Unknown	1.01 (0.94, 1.09)	1.01 (0.91, 1.12)	1.75 (1.54, 1.99)***	1.26 (1.20, 1.33)***
Quantity for sale (g)				
1	1.25 (1.19, 1.32)***	1.26 (1.17, 1.35)***	1.39 (1.32, 1.46)***	1.31 (1.27, 1.36)***
2	1.27 (1.17, 1.39)***	1.19 (1.13, 1.27)***	1.29 (1.21, 1.39)***	1.14 (1.06, 1.22)***
5	ref	ref	ref	ref
10	0.66 (0.64, 0.69)***	0.84 (0.81, 0.87)***	0.84 (0.81, 0.87)***	0.74 (0.72, 0.76)***
25	0.43 (0.42, 0.45)***	0.72 (0.69, 0.75)***	0.60 (0.57, 0.62)***	0.48 (0.47, 0.49)***
50	0.36 (0.34, 0.37)***	0.64 (0.61, 0.67)***	0.53 (0.51, 0.55)***	0.41 (0.40, 0.43)***
100	0.30 (0.29, 0.32)***	0.51 (0.49, 0.53)***	0.45 (0.43, 0.47)***	0.33 (0.32, 0.34)***
250	0.25 (0.24, 0.27)***	0.41 (0.39, 0.43)***	0.35 (0.33, 0.36)***	0.25 (0.24, 0.26)***
500	0.22 (0.21, 0.23)***	0.27 (0.25, 0.28)***	0.30 (0.29, 0.32)***	0.23 (0.22, 0.23)***
1000	0.19 (0.18, 0.20)***	0.16 (0.15, 0.17)***	0.25 (0.24, 0.26)***	0.17 (0.16, 0.17)***

*Note:* The estimates in this table were computed from an omnibus model for price per gram by fitting an interaction effect for each of these nitazenes and all other nitazenes with their availability for delivery to Australia, geographical region of origin, quantity offered for purchase and the time of snapshot using the thin‐plate regression spline. The price ratios are presented with respect to the referent group for each characteristic within each nitazene type. Etonitazene was included within the other nitazenes group in this analysis as there were too few etonitazene listings.

Abbreviation: CI, confidence interval.

* Statistical significance at *p* < 0.05.

*** Statistical significance at *p* < 0.001.

#### 3.2.2 Region of Origin

Compared with nitazenes originating from Asia (median 38.00 USD per gram), those from North America, Europe and Oceania were 1.04 (95% CI = 1.01, 1.07), 1.25 (95% CI = 1.20, 1.31) and 1.10 (95% CI = 1.06, 1.14) times more expensive, respectively (median 77.30, 87.50 and 64.00 USD per gram, respectively) (Table 1). The direction of association was similar for protonitazene and metonitazene listings but it was not statistically significant for unknown geographical origin (metonitazene only) and for Oceania. For isotonitazene, listings originating from North America (price ratio = 0.91; 95% CI = 0.88, 0.93) and Oceania (price ratio = 0.80; 95% CI = 0.70, 0.91) were significantly cheaper compared with those originating from Asia, while those originating from Europe were more expensive (price ratio = 1.81; 95% CI = 1.60, 2.04) (Table 2).

#### 3.2.3 Quantity

Nitazenes sold in larger quantities were significantly lower in price than those sold in smaller quantities (Tables 1 and 2), with a median price of 124.42 USD per gram for 1 g of nitazene compared to 16.50 USD per gram for 1 kg of nitazene. Compared with metonitazene and protonitazene, isotonitazene had the highest median price across the quantities we have analysed in the study (Table E in Appendix III). The price of isotonitazene ranged from a median of 212.64 USD per gram for 1 g to 23.50 USD per gram for 1 kg. The median price of metonitazene and protonitazene was 90.00 USD per gram for 1 g, respectively and 13.80 and 18.80 USD per gram for 1 kg of metonitazene and protonitazene, respectively. There were too few etonitazene listings identified on the cryptomarkets for a separate analysis.

#### 3.2.4 Time

**FIGURE 1:**
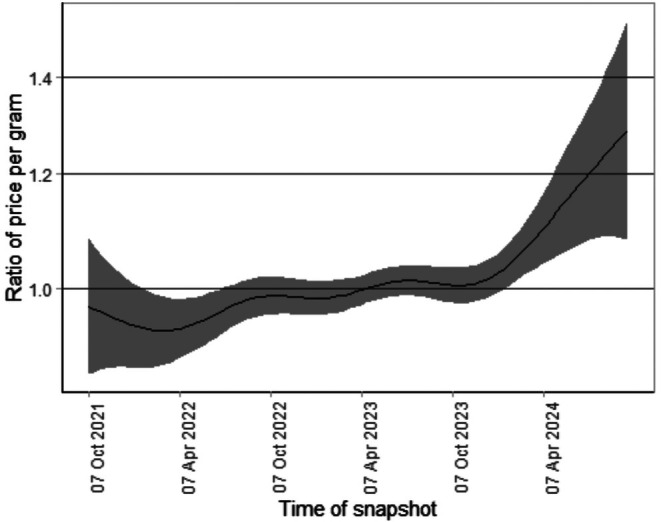
Estimated trend in price per gram of nitazene powders. This is the smoothed trend line estimated in the generalised additive model (see Section 2) fitted to the cryptomarket listings data collected twice monthly from October 2021 to September 2024. The trend in price per gram is expressed as a ratio of the estimated price per gram at the time point where the ratio is one, i.e., the estimated price at around 7 April 2023 snapshot is the reference point. It is presented on a logarithmic scale on the *y*‐axis. The grey band shows the 95% confidence interval.

**FIGURE 2:**
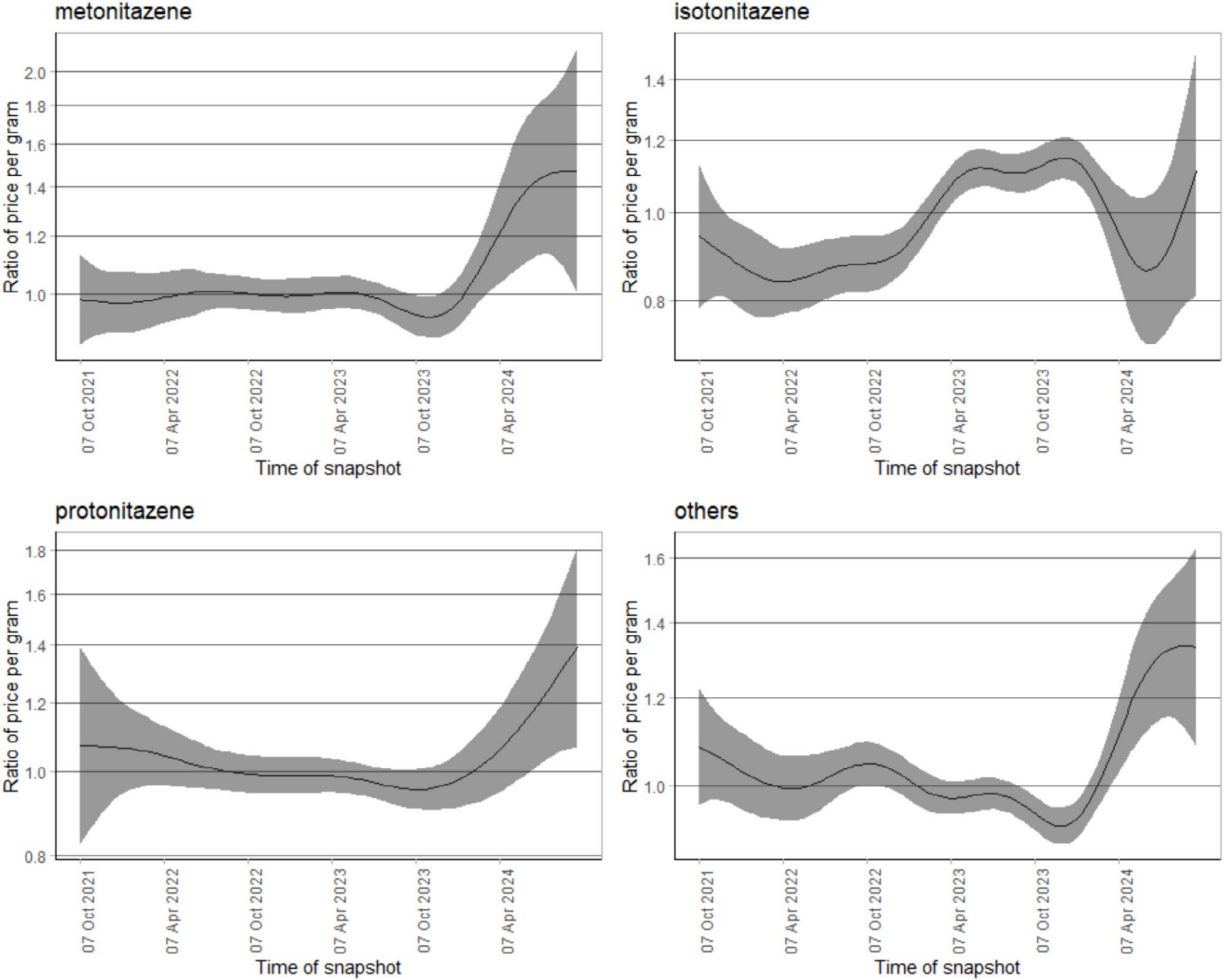
Estimated trend in price per gram of metonitazene, isotonitazene, protonitazene, and other nitazenes. The trend in price per gram on the *y*‐axis is expressed as a ratio of the estimated price per gram at the point where the ratio is one. It is presented on a logarithmic scale on the *y*‐axis. The grey band shows the 95% confidence interval.

There was a significant non‐linear relationship of price with snapshot period (*p* < 0.001) with a trend of increasing price from the beginning of the year 2024 (Figure 1). This trend was similar for protonitazene and metonitazene (Figure 2). For isotonitazene, the price increased from around the beginning of the year 2023, then dropped momentarily from the beginning of 2024 before recovering in the middle of the year until the end of the study. Table F in Appendix III shows that isotonitazene had the highest median price compared with metonitazene and protonitazene at both small (1–10 g) and larger (> 10 g) quantities in each of the 3 years in this study.

### 3.3 Pricing on Surface Web Shops

The median price for the minimum quantity of the four nitazenes of interest was 32.30 USD per gram for metonitazene and 35.00 USD per gram for the other three nitazenes and the median price for the maximum quantity was between 9.00 USD per gram for metonitazene and 14.50 USD per gram for etonitazene (Table 3). Among the prices for the minimum quantity available for purchase, the median price for 5 and 10 g of any of the four nitazenes was 57.00 and 29.50 USD per gram, respectively. Among the prices for the maximum quantity available for purchase, the median price for 100 g of any of these nitazenes was 16.00 USD per gram; cheaper prices were available for larger quantities.

**TABLE 3:** Prices (USD per gram) of the four key nitazenes available for purchase on surface net shops.

	Number of listings	Price in USD per gram	
		Min. quantity for sale	Max. quantity for sale
		Median price (Q1, Q3)	Median price (Q1, Q3)
Nitazene name			
Metonitazene	5	32.30 (30.00, 37.00)	9.00 (4.99, 10.20)
Isotonitazene	7	35.00 (30.51, 47.00)	11.90 (5.78, 14.90)
Protonitazene	7	35.00 (28.00, 52.50)	11.00 (7.85, 15.50)
Etonitazene	6	35.00 (30.50, 45.50)	14.50 (7.39, 17.52)
Quantity for sale			
5 g	11	57.00 (42.50, 78.50)	—
10 g	14	29.50 (26.00, 35.00)	—
100 g	2	—	16.00 (14.50, 17.50)
500 g	4	—	10.60 (8.99, 12.68)
1000 g	11	—	12.00 (5.85, 17.25)
2000 g	4	—	5.70 (4.87, 5.70)
≥ 5000 g	4	—	11.90 (9.75, 14.35)
Overall	25	35.00 (29.00, 49.00)	11.00 (5.70, 17.00)

*Note:* Q1 = 1st quartile; Q3 = 3rd quartile.

## 4 Discussion

This is the first study to examine the price of nitazenes available for purchase via cryptomarkets and surface web shops. Price varied markedly based on nitazene type and quantity, although prices were lower on surface web shops than on cryptomarkets. Study by country showed that listings available to be shipped to Australia were more expensive, as were those that advertised as shipping from Oceania, North America and Europe (relative to Asia). These findings provide new insight into the online supply and pricing dynamics of nitazenes, highlighting key factors that may influence their accessibility and potential for harm.

Overall, the price of listed nitazenes increased across the study period (controlling for quantity), with the most notable spike occurring from early 2024 onwards. This was largely consistent across the different types of nitazenes, except for isotonitazene, where more fluctuation was observed. While the exact drivers of this overall trend of increasing price remain unclear, one possible explanation is growing awareness of nitazenes. The increasing public health burden [7, 28]—alongside an increase in associated media coverage—may have contributed to an increase in demand. At the same time, greater recognition of nitazene‐related overdoses and deaths [28, 38] may have prompted increased law enforcement scrutiny, thereby elevating the risks for manufacturers and sellers and contributing to higher prices. Indeed, elsewhere we have reported a decline in the number of nitazene cryptomarket listings since 2024, suggesting nitazenes may be less available on these platforms [32]. These interpretations, however, are largely speculative and continued monitoring is essential to better understand these evolving trends.

On both cryptomarkets and surface web shops there was a tendency towards a higher median price per gram for nitazenes of greater potency (e.g., isotonitazene) [2] and lower price per gram for greater quantity purchases. This reflects well‐established patterns in illicit drug markets, in which potency and purchase volume are key determinants of unit cost [39, 40]. To date, there is no documented retail or ‘street price’ for nitazenes; however, these online prices per gram were generally lower than the reported median price per gram of heroin in Australia in 2024 ($325.00 AUD; ~$209.00 USD) [41].

All nitazenes, except isotonitazene, were less expensive when originating in Asia than from North America, Europe or Oceania. More than two out of every three listings originated in Asia, indicating greater production capacity within Asia compared to these regions which, in turn, may facilitate lower prices [28], although it is important to note that number of listings originating from Asia (and specifically China) has declined since 2024 [32]. On 1 July 2025, China placed nitazene analogues under national control; the impact of this scheduling on availability and price has yet to be established [8]. Strict drug laws and stronger enforcement in North America, Europe and Oceania may increase the risks for producers and sellers [38, 42], contributing to the higher prices observed in this study. Similarly, listings offering delivery to Australia were more expensive than those that did not, although this varied by nitazene type. For metonitazene, prices did not differ significantly between listings that offered delivery to Australia and those that did not, while for isotonitazene, prices were lower among listings offering delivery to Australia. These findings could reflect the overall higher cost of isotonitazene and the fact that listings originating from Oceania were cheaper than those originating from Asia, a pattern not observed for other nitazenes. In contrast and consistent with the broader trend, listings for protonitazene and ‘other nitazenes’ that offered delivery to Australia were more expensive than those that did not. This likely reflects Australia's greater geographic isolation and relatively strong border enforcement, which, again, can increase the risks and costs to sellers and traffickers [38]. Indeed, Australian consumers typically pay among the highest retail prices for illicit drugs globally, which can incentivise trafficking and organised crime networks to target the Australian market [43].

Nitazene listings on cryptomarkets tended to be available in smaller quantities (e.g., 1 g, compared to a minimum quantity of 5 g on surface web shops) and were more expensive than listings on the surface web. This price disparity may reflect several factors, including higher operational costs and risks (e.g., exit scams) associated with the more clandestine nature of cryptomarkets. Most cryptomarkets rely on an escrow purchasing system and charge the seller administrative fees for this service which invariably increases the price of listed goods [44]. Further, access to cryptomarkets requires the use of the Tor browser and requires payment via cryptocurrencies which may carry their own additional exchange fees [28]. It is also possible that surface web vendors face fewer reputational risks, as many operate with limited accountability and tend to disappear quickly, an observation supported by earlier monitoring work showing that these stores often pop in and out of existence [45]. In any case, the availability of nitazenes at lower prices but with higher minimum purchase quantities via more easily accessible surface web shops is concerning and underscores the importance of proactive harm reduction strategies such as take‐home naloxone [13], nitazene test strips [46] and the expansion of drug checking services [47].

### 4.1 Limitations

Findings should be interpreted with caution as they represent an approximation of nitazene availability on cryptomarkets and surface web shops. Active markets may have been missed due to identification lags. Only English language markets were analysed, with other data suggesting non‐Western markets (particularly Russian markets) comprise a large proportion of the cryptomarket environment [48]. One of the largest Western markets, ‘Abacus’ was also excluded due to onerous security measures that made data collection unfeasible, noting that this market closed in July 2025. While these limitations affect coverage, analysis of high‐volume markets provides a reasonable proxy for broader trends. Relatedly, we cannot infer sales from these data; future work studying vendor ratings and buyer feedback could be used to establish a proxy measure of purchasing across nitazene type, quantity and price point [49].

Finally, this study was restricted to the available data for each listing, all of which is self‐reported by vendors. The actual value of a drug purchase reflects an interplay between the quantity, the price and the purity; previous research has shown that study of price unadjusted for purity, as was the case in the present study, can reduce sensitivity in detecting major market shifts [50]. The diversity in characteristics of listings (e.g., quantity for purchase, geographical origin and destination) over a relatively long period of time also did not allow us to directly compare nitazene types in our study or study the association of price with prevalence over time or potency. Study of drug samples purchased from cryptomarkets and submitted to drug checking services may be a way to obtain all necessary information for such analysis [29]. Such work could also assist in objectively confirming listing information, including substance contents. Indeed, previous research has identified discrepancy between nitazenes sold via cryptomarkets as advertised versus analytically confirmed [29], as well as mixed findings across drug types regarding whether adulteration is more common in drugs purchased from online versus offline markets [51].

## 5 Conclusion

Pricing of nitazenes on cryptomarkets and surface web markets was highly variable and influenced by factors such as quantity, type and geographic origin. They were advertised cheaper on surface web shops than cryptomarkets. This combination of relative low‐cost, high potency and sustained availability of nitazenes through online markets represents a growing public health concern in Australia and internationally [13, 14, 15, 52]. These findings reinforce the importance of strong data monitoring systems that enable timely, sensitive detection of harms alongside proactive harm reduction strategies.

## Author Contributions

M.J.B., R.B., A.P. and N.M. conceived the study. All authors had input on the study design. V.S. and N.M. prepared the data for analysis. N.M. conducted analyses with support from V.S. N.M., A.P., R.B., R.S., M.J.B. and C.G. drafted the manuscript. All authors had input on revising the manuscript critically for important intellectual content. All authors approved the final version to be published. Each author certifies that their contribution to this work meets the standards of the International Committee of Medical Journal Editors.

## Funding

Drug Trends, the National Drug and Alcohol Research Centre and the National Drug Research Institute are funded by the Australian Government Department of Health, Disability and Ageing under the Drug and Alcohol Program. M.J.B. (#2042605), A.P. (#1174630) and R.S. (#1197241) are supported by National Health and Medical Research Council Investigator Fellowships.

## Conflicts of Interest

The authors declare no conflicts of interest.

## Supporting information


**Appendix I:** STROBE Checklist.
**Table A**. STROBE Statement—Checklist of items that should be included in reports of cross‐sectional studies.
**Appendix II:** Additional information for methods.
**Figure A**. Gantt chart of markets included in the study.
**Table B**. List of specific nitazenes and terms for identifying specific nitazenes.
**Table C**. Terms for identifying form of drug.
**Table D**. Identified country of origin (in descending order of frequency) and their region of origin in this study.
**Appendix III:** Additional information for results.
**Figure B**. Flowchart of exclusions for cryptomarket pricing data.
**Table E**. Median prices in USD per gram (and frequency) of the key nitazenes available for purchase on cryptomarkets by quantity for sale.
**Table F**. Median prices in USD per gram (and frequency) of the key nitazenes available for purchase in small (1–10 g) and larger quantities (> 10 g) on cryptomarkets by availability for delivery to Australia, region of origin and year.

## Data Availability

The data that support the findings of this study are available on request from the corresponding author. The data are not publicly available due to privacy or ethical restrictions.
