# Supplementary material for: Price of Nitazenes for Sale on Cryptomarkets and Surface Web Shops
Source: Drug Alcohol Rev. 2026 Jul 8;45(5):e70202. doi: 10.1111/dar.70202 (PMC13346510; doi:10.1111/dar.70202)
Supplement: Supplementary file 1 — Appendix I: STROBE Checklist. Table A. STROBE Statement—Checklist of items that should be included in reports of cross‐sectional studies. Appendix II: Additional information for methods. Figure A. Gantt chart of markets included in the study. Table B. List of specific nitazenes and terms for identifying specific nitazenes. Table C. Terms for identifying form of drug. Table D. Identified country of origin (in descending order of frequency) and their region of origin in this study. Appendix III: Additional information for results. Figure B. Flowchart of exclusions for cryptomarket pricing data. Table E. Median prices in USD per gram (and frequency) of the key nitazenes available for purchase on cryptomarkets by quantity for sale. Table F. Median prices in USD per gram (and frequency) of the key nitazenes available for purchase in small (1–10 g) and larger quantities (> 10 g) on cryptomarkets by availability for delivery to Australia, region of origin and year. [file DAR-45-0-s001.docx]

# Supplementary material

## Appendix I. STROBE Checklist

### **Table A.** STROBE Statement—Checklist of items that should be included in reports of cross-sectional studies.

|  | Item No | Recommendation | Complete |
| --- | --- | --- | --- |
| **Title and abstract** | 1 | (*a*) Indicate the study’s design with a commonly used term in the title or the abstract | ✓ |
|  |  | (*b*) Provide in the abstract an informative and balanced summary of what was done and what was found | ✓ |
| Introduction | | |  |
| Background/rationale | 2 | Explain the scientific background and rationale for the investigation being reported | ✓ |
| Objectives | 3 | State specific objectives, including any prespecified hypotheses | ✓ |
| Methods | | |  |
| Study design | 4 | Present key elements of study design early in the paper | ✓ |
| Setting | 5 | Describe the setting, locations, and relevant dates, including periods of recruitment, exposure, follow-up, and data collection | ✓ |
| Participants | 6 | (*a*) Give the eligibility criteria, and the sources and methods of selection of participants | ✓ |
| Variables | 7 | Clearly define all outcomes, exposures, predictors, potential confounders, and effect modifiers. Give diagnostic criteria, if applicable | ✓ |
| Data sources/ measurement | 8* | For each variable of interest, give sources of data and details of methods of assessment (measurement). Describe comparability of assessment methods if there is more than one group | ✓ |
| Bias | 9 | Describe any efforts to address potential sources of bias | ✓ |
| Study size | 10 | Explain how the study size was arrived at | N.A. (given secondary data sources) |
| Quantitative variables | 11 | Explain how quantitative variables were handled in the analyses. If applicable, describe which groupings were chosen and why | ✓ |
| Statistical methods | 12 | (*a*) Describe all statistical methods, including those used to control for confounding | ✓ |
|  |  | (*b*) Describe any methods used to examine subgroups and interactions | N.A. |
|  |  | (*c*) Explain how missing data were addressed | N.A. |
|  |  | (*d*) If applicable, describe analytical methods taking account of sampling strategy | ✓ |
|  |  | (*e*) Describe any sensitivity analyses | ✓ |
| Results | | |  |
| Participants | 13* | (a) Report numbers of individuals at each stage of study—eg numbers potentially eligible, examined for eligibility, confirmed eligible, included in the study, completing follow-up, and analysed | ✓ |
|  |  | (b) Give reasons for non-participation at each stage | N.A. |
|  |  | (c) Consider use of a flow diagram | N.A. |
| Descriptive data | 14* | (a) Give characteristics of study participants (eg demographic, clinical, social) and information on exposures and potential confounders | ✓ |
|  |  | (b) Indicate number of participants with missing data for each variable of interest | N.A. |
| Outcome data | 15* | Report numbers of outcome events or summary measures | ✓ |
| Main results | 16 | (*a*) Give unadjusted estimates and, if applicable, confounder-adjusted estimates and their precision (eg, 95% confidence interval). Make clear which confounders were adjusted for and why they were included | ✓ |
|  |  | (*b*) Report category boundaries when continuous variables were categorized | N.A. |
|  |  | (*c*) If relevant, consider translating estimates of relative risk into absolute risk for a meaningful time period | N.A. |
| Other analyses | 17 | Report other analyses done—eg analyses of subgroups and interactions, and sensitivity analyses | ✓ |
| Discussion | | |  |
| Key results | 18 | Summarise key results with reference to study objectives | ✓ |
| Limitations | 19 | Discuss limitations of the study, taking into account sources of potential bias or imprecision. Discuss both direction and magnitude of any potential bias | ✓ |
| Interpretation | 20 | Give a cautious overall interpretation of results considering objectives, limitations, multiplicity of analyses, results from similar studies, and other relevant evidence | ✓ |
| Generalisability | 21 | Discuss the generalisability (external validity) of the study results | ✓ |
| Other information | | |  |
| Funding | 22 | Give the source of funding and the role of the funders for the present study and, if applicable, for the original study on which the present article is based | ✓ |

## Appendix II. Additional information for methods

The Gantt chart below shows the available cryptomarket data for all drug listings from October 2021 to September 2024, noting that we limited our study to English-language markets. Televend was excluded because it is a variant on the traditional cryptomarket and it was only available in the very first scrape in October 2021. Abacus was excluded because of the labour-intensive nature of the scrape preventing regular scrapes from being undertaken (and has since closed as of July 2025).

### **Figure A.** Gantt chart of markets included in the study.


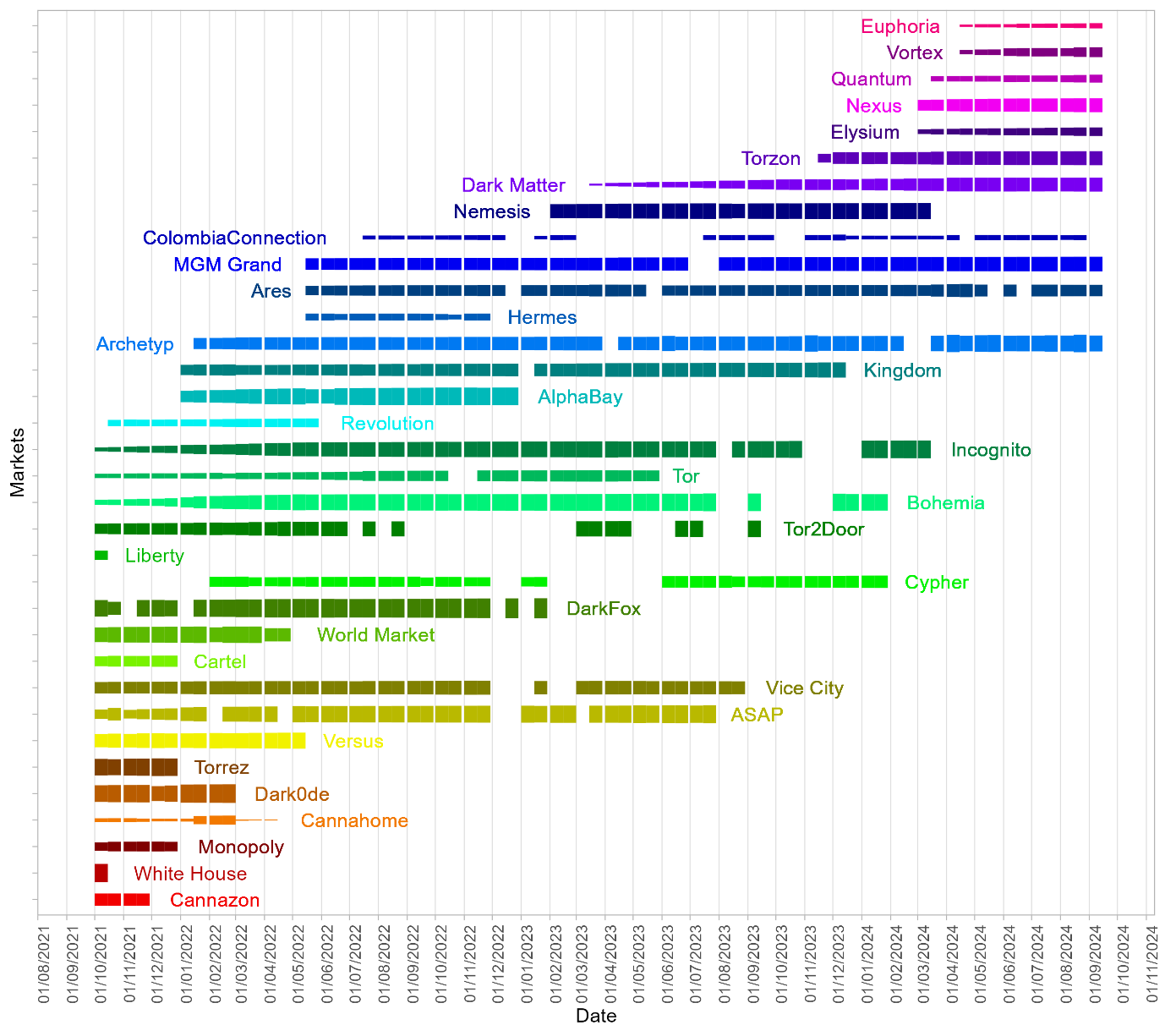


Note: There are short breaks at the end of each month because the twice-monthly snapshots were based on 2-week intervals starting on the 1^st^ and 15^th^ of each month, i.e., days of the month after the 28^th^ were excluded from monitoring. Extended breaks indicate periods where market crawling was not achievable or incomplete. The height of the bar is proportional to the log of the number of all drug listings observed in each snapshot for each market.

### **Table B.** List of specific nitazenes and terms for identifying specific nitazenes

| **Specific nitazene** | **Identification** |
| --- | --- |
| Butonitazene | Includes those mis-spelt as ‘sthlmbutonitazene’. |
| Etomethazene | Alternative terms not identified. |
| Etonitazene | Includes those mis-spelt as ‘etonitazen’ and ‘etonitaze’. |
| Fluonitazene | Alternative terms not identified. Also known as ‘flunitazene’ but the synonym was not identified in the cryptomarket listings. |
| Isotonitazene | Includes ‘isonitazene’ and the mis-spelt names, ‘isotoniazene’ and ‘sotonitazene’. |
| Metonitazene | Includes those mis-spelt as ‘metanitazene’ and ‘metronitazene’. |
| Protonitazene | Includes ‘propoxynitazene’, ‘pronitazene’, and those mis-spelt, e.g., ‘rotonitazene’. |
| Etodesnitazene | Includes ‘etazene’ and those mis-spelt as ‘etazen’. |
| Metodesnitazene | Includes ‘metazene’. |
| Protodesnitazene | Alternative terms not identified. |
| Etonitazepyne^1^ | Includes N-pyrrolidino etonitazene identified with the term ‘pyrrolidino etonitazene’. |
| Isotonitazepyne^1^ | Includes N-pyrrolidino isotonitazene identified with the term ‘pyrrolidino isotonitazene’ and those mis-spelt name as ‘isotonitapyne’. |
| Metonitazepyne^1^ | Includes N-pyrrolidino metonitazene identified with the term ‘pyrrolidino metonitazene’. |
| Protonitazepyne^1^ | Includes N-pyrrolidino protonitazene identified with the term ‘pyrrolidino protonitazene’. |
| Etonitazepipne^2^ | Also known as ‘N-piperidino etonitazene’ or ‘N-piperidinyl etonitazene’, and identified with the terms ‘piperidino etonitazene’ or ‘piperidinyl etonitazene’, respectively. |
| N-desethyl etonitazene | Identified with the terms ‘desethyl etonitazene’ and ‘desethyletonitazene’. Also known as ‘noretonitazene’ but the synonym was not identified in the cryptomarket listings. |
| N-desethyl isotonitazene | Identified with the terms ‘desethyl isotonitazene’ and ‘desethylisotonitazene’. Includes those mis-spelt as ‘desethyl isotoniazene’. Also known as ‘norisotonitazene’ but the synonym was not identified in the cryptomarket listings. |
| N-desethyl protonitazene | Identified with the terms ‘desethyl protonitazene’ and ‘desethylprotonitazene’. |

Note: ^1^Potential nitazene listings were searched for the substrings ‘pyr’ and ‘pyne’ for the nitazenes ending with “-azepyne” to verify that these nitazenes including mis-spelt ones were comprehensively identified. ^2^Potential nitazene listings were searched for the substrings ‘pip’ and ‘dinyl’ for the nitazenes ending with “-azepipne” to verify that these nitazenes including mis-spelt ones were comprehensively identified.

### **Table C.** Terms for identifying form of drug.

| **Form of drug** | **Example of terms in drug title** | **Example of terms for quantity (in a quantity field or in listing title)** |
| --- | --- | --- |
| Powder | “powder” | “mcg”, “mg”, “g”, “gram”, “kg”, and with no “x” to indicate multiples (e.g., for pills or tablets) |
| Pill/tablet/press/capsule | “pills”, “tabs”, “caps*”, “percocet”, “m30” | “pill”, “tb”, “cap”, “tab” |
| Liquid | “spray” | “ml*” |
| Blotter | “blot*”^1^ | -- |
| Unknown | (None of above found) | -- |

Note: ^1^ The listing titles and quantity fields were converted to lowercase before matching on the terms. The listings with an unknown form of the drug were examined and the author (NM) did not find any information that might be used to deduce the form of the drug. The term “blot*” for blotter overrides the classification for pill/tablet/press/capsule because the terms “tab” or “tb” can be the quantity unit for blotters.

The listings with an unknown form of the drug were examined and the author (NM) did not find any information that might be used to deduce the form of the drug.

### **Table D.** Identified country of origin (in descending order of frequency) and their region of origin in this study.

| **Region of origin** | **Identified country (or region^1^) of origin** |
| --- | --- |
| Asia | China, Hong Kong, Afghanistan, Taiwan, Asia^1^, Korea, Singapore |
| North America | United States, Canada, North America^1^ |
| Europe | United Kingdom, Czech Republic, Europe^1^, Germany, Sweden, Switzerland, Albania, France, Finland, Netherlands, Austria |
| Oceania | Australia, New Zealand |
| Unknown | Unknown or missing |

Note: ^1^ Only the continental region was specified in these listings.

## Appendix III. Additional information for results

### **Figure B.** Flowchart of exclusions for cryptomarket pricing data


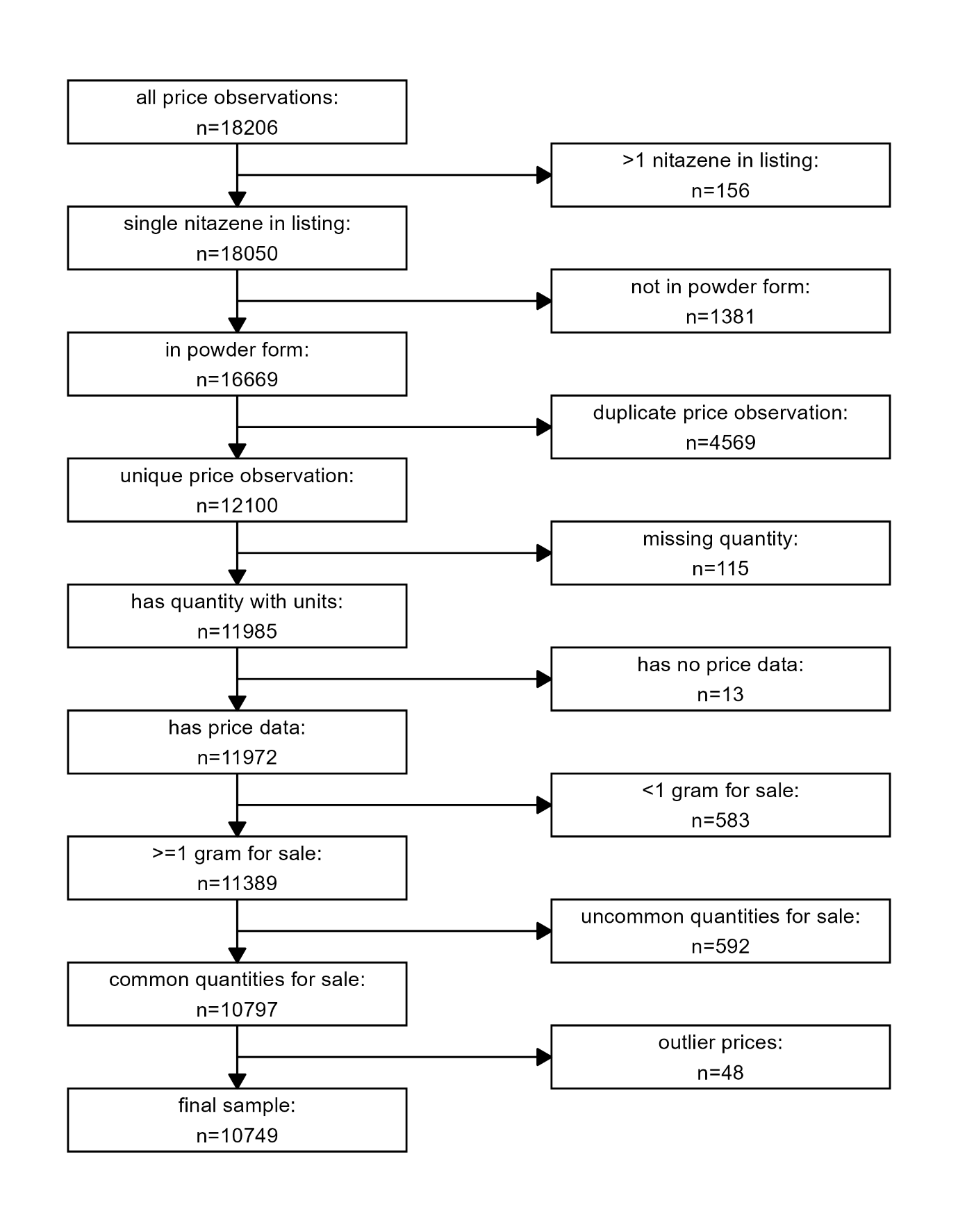


### **Table E.** Median prices in USD per gram (and frequency) of the key nitazenes available for purchase on cryptomarkets by quantity for sale

|  | **Metonitazene** | | **Isotonitazene** | | **Protonitazene** | |
| --- | --- | --- | --- | --- | --- | --- |
|  | **N** | **Median (Q1, Q3)^a^** | **N** | **Median (Q1, Q3)^a^** | **N** | **Median (Q1, Q3)^a^** |
| **Quantity for sale (grams)** | | | | | | |
| 1 | 124 | 90.00 (90.00, 140.00) | 62 | 212.64 (130.03, 240.00) | 113 | 90.00 (90.00, 100.00) |
| 2 | 35 | 87.50 (85.00, 87.50) | 86 | 95.00 (87.50, 225.00) | 68 | 87.50 (87.50, 87.50) |
| 5 | 382 | 64.00 (64.00, 77.20) | 495 | 86.00 (77.04, 127.15) | 365 | 70.00 (70.00, 74.00) |
| 10 | 463 | 45.00 (40.00, 57.00) | 327 | 78.00 (60.00, 140.00) | 262 | 60.00 (60.00, 65.00) |
| 25 | 190 | 24.80 (24.00, 41.50) | 224 | 112.00 (56.00, 150.00) | 180 | 36.00 (36.00, 38.00) |
| 50 | 182 | 22.00 (22.00, 40.00) | 197 | 100.00 (52.00, 144.00) | 197 | 32.00 (32.00, 32.00) |
| 100 | 192 | 19.50 (16.00, 35.00) | 201 | 80.00 (49.00, 97.00) | 179 | 29.00 (29.00, 29.00) |
| 250 | 125 | 18.40 (18.40, 25.60) | 142 | 71.60 (56.00, 75.70) | 137 | 25.20 (24.00, 25.20) |
| 500 | 169 | 17.00 (12.00, 17.00) | 121 | 42.00 (38.00, 50.00) | 152 | 22.00 (17.00, 22.00) |
| 1000 | 161 | 13.80 (13.80, 15.70) | 135 | 23.50 (21.00, 24.00) | 162 | 18.80 (14.00, 18.80) |
| **Overall** | 2023 | 40.00 (18.85, 64.00) | 1990 | 80.00 (52.00, 120.00) | 1815 | 38.00 (25.20, 70.00) |

### **Table F.** Median prices in USD per gram (and frequency) of the key nitazenes available for purchase in small (1-10 grams) and larger quantities (>10 grams) on cryptomarkets by availability for delivery to Australia region of origin and year

|  | **Metonitazene** | | **Isotonitazene** | | **Protonitazene** | |
| --- | --- | --- | --- | --- | --- | --- |
|  | **N** | **Median (Q1, Q3)^a^** | **N** | **Median (Q1, Q3)^a^** | **N** | **Median (Q1, Q3)^a^** |
| **For 1-10 grams quantities** | | | | | | |
| **Delivery to Australia** | | | | | | |
| Yes | 941 | 64.00 (45.00, 78.00) | 828 | 90.00 (70.00, 172.00) | 744 | 70.00 (60.00, 81.00) |
| No | 63 | 50.00 (46.00, 72.50) | 142 | 86.00 (80.00, 87.20) | 64 | 74.00 (65.00, 74.00) |
| **Region of origin** | | | | | | |
| Asia | 555 | 64.00 (59.77, 83.00) | 333 | 80.00 (70.00, 200.00) | 606 | 70.00 (60.00, 77.50) |
| North America | 271 | 48.00 (42.00, 76.00) | 434 | 94.00 (74.00, 160.00) | 150 | 81.00 (74.00, 87.50) |
| Europe | 88 | 50.00 (40.00, 160.00) | 105 | 87.50 (77.00, 123.68) | 16 | 190.00 (150.02, 290.00) |
| Oceania | 40 | 40.00 (40.00, 41.25) | 20 | 94.00 (2.00, 94.00) | 1 | 301.50 (--) |
| Unknown | 50 | 65.00 (48.00, 65.00) | 78 | 80.00 (62.00, 86.00) | 35 | 74.00 (74.00, 74.00) |
| **Snapshot period** | | | | | | |
| Oct 2021 - Sep 2022 | 429 | 60.00 (40.00, 65.00) | 242 | 78.00 (60.00, 86.00) | 189 | 70.00 (60.00, 80.00) |
| Oct 2022 - Sep 2023 | 465 | 64.00 (45.00, 78.00) | 457 | 91.00 (73.93, 160.00) | 447 | 70.00 (60.00, 80.00) |
| Oct 2023 - Sep 2024 | 110 | 76.00 (70.00, 110.00) | 271 | 120.00 (85.01, 180.00) | 172 | 75.00 (62.31, 87.50) |
| **For >10 grams quantities** | | | | | | |
| **Delivery to Australia** | | | | | | |
| Yes | 993 | 19.40 (15.94, 24.80) | 994 | 71.20 (42.00, 100.00) | 950 | 27.96 (22.00, 36.00) |
| No | 26 | 12.00 (12.00, 17.82) | 26 | 37.73 (33.00, 48.50) | 57 | 20.00 (16.20, 25.86) |
| **Region of origin** | | | | | | |
| Asia | 857 | 19.50 (17.00, 24.00) | 599 | 52.00 (42.00, 95.00) | 944 | 27.96 (22.00, 36.00) |
| North America | 74 | 34.00 (17.80, 80.00) | 333 | 80.00 (38.00, 100.00) | 19 | 23.21 (17.63, 27.38) |
| Europe | 12 | 24.80 (24.80, 24.80) | 80 | 94.58 (74.89, 100.71) | 13 | 12.74 (11.98, 22.07) |
| Oceania | 14 | 16.00 (16.00, 16.00) | 0 | -- | 11 | 17.00 (16.50, 30.60) |
| Unknown | 62 | 12.00 (12.00, 12.00) | 8 | 26.95 (20.90, 33.00) | 20 | 17.10 (15.00, 20.00) |
| **Snapshot period** | | | | | | |
| Oct 2021 - Sep 2022 | 300 | 19.50 (15.99, 34.00) | 174 | 49.00 (26.00, 56.00) | 185 | 29.00 (22.00, 36.00) |
| Oct 2022 - Sep 2023 | 597 | 18.60 (16.00, 24.00) | 541 | 66.71 (42.00, 100.86) | 717 | 27.96 (22.00, 32.00) |
| Oct 2023 - Sep 2024 | 122 | 18.60 (15.44, 35.00) | 305 | 84.00 (50.00, 112.00) | 105 | 18.00 (15.00, 27.16) |

Note: ^a^ Q1 = 1^st^ quartile; Q3 = 3^rd^ quartile.
